# Assessment of a Clinical Trial–Derived Survival Model in Patients With Metastatic Castration-Resistant Prostate Cancer

**DOI:** 10.1001/jamanetworkopen.2020.31730

**Published:** 2021-01-22

**Authors:** Jean Coquet, Nicolas Bievre, Vincent Billaut, Martin Seneviratne, Christopher J. Magnani, Selen Bozkurt, James D. Brooks, Tina Hernandez-Boussard

**Affiliations:** 1 Department of Medicine, Stanford University School of Medicine, Stanford, California; 2 Department of Statistics, Stanford University, Stanford, California; 3 Department of Biomedical Data Science, Stanford University, Stanford, California; 4 Stanford University School of Medicine, Stanford, California; 5 Department of Urology, Stanford University School of Medicine, Stanford, California; 6 Stanford Cancer Institute, Stanford University School of Medicine, Stanford, California; 7 Department of Surgery, Stanford University School of Medicine, Stanford, California

## Abstract

**Importance:**

Randomized clinical trials (RCTs) are considered the criterion standard for clinical evidence. Despite their many benefits, RCTs have limitations, such as costliness, that may reduce the generalizability of their findings among diverse populations and routine care settings.

**Objective:**

To assess the performance of an RCT-derived prognostic model that predicts survival among patients with metastatic castration-resistant prostate cancer (CRPC) when the model is applied to real-world data from electronic health records (EHRs).

**Design, Setting, and Participants:**

The RCT-trained model and patient data from the RCTs were obtained from the Dialogue for Reverse Engineering Assessments and Methods (DREAM) challenge for prostate cancer, which occurred from March 16 to July 27, 2015. This challenge included 4 phase 3 clinical trials of patients with metastatic CRPC. Real-world data were obtained from the EHRs of a tertiary care academic medical center that includes a comprehensive cancer center. In this study, the DREAM challenge RCT-trained model was applied to real-world data from January 1, 2008, to December 31, 2019; the model was then retrained using EHR data with optimized feature selection. Patients with metastatic CRPC were divided into RCT and EHR cohorts based on data source. Data were analyzed from March 23, 2018, to October 22, 2020.

**Exposures:**

Patients who received treatment for metastatic CRPC.

**Main Outcomes and Measures:**

The primary outcome was the performance of an RCT-derived prognostic model that predicts survival among patients with metastatic CRPC when the model is applied to real-world data. Model performance was compared using 10-fold cross-validation according to time-dependent integrated area under the curve (iAUC) statistics.

**Results:**

Among 2113 participants with metastatic CRPC, 1600 participants were included in the RCT cohort, and 513 participants were included in the EHR cohort. The RCT cohort comprised a larger proportion of White participants (1390 patients [86.9%] vs 337 patients [65.7%]) and a smaller proportion of Hispanic participants (14 patients [0.9%] vs 42 patients [8.2%]), Asian participants (41 patients [2.6%] vs 88 patients [17.2%]), and participants older than 75 years (388 patients [24.3%] vs 191 patients [37.2%]) compared with the EHR cohort. Participants in the RCT cohort also had fewer comorbidities (mean [SD], 1.6 [1.8] comorbidities vs 2.5 [2.6] comorbidities, respectively) compared with those in the EHR cohort. Of the 101 variables used in the RCT-derived model, 10 were not available in the EHR data set, 3 of which were among the top 10 features in the DREAM challenge RCT model. The best-performing EHR-trained model included only 25 of the 101 variables included in the RCT-trained model. The performance of the RCT-trained and EHR-trained models was adequate in the EHR cohort (mean [SD] iAUC, 0.722 [0.118] and 0.762 [0.106], respectively); model optimization was associated with improved performance of the best-performing EHR model (mean [SD] iAUC, 0.792 [0.097]). The EHR-trained model classified 256 patients as having a high risk of mortality and 256 patients as having a low risk of mortality (hazard ratio, 2.7; 95% CI, 2.0-3.7; log-rank *P* < .001).

**Conclusions and Relevance:**

In this study, although the RCT-trained models did not perform well when applied to real-world EHR data, retraining the models using real-world EHR data and optimizing variable selection was beneficial for model performance. As clinical evidence evolves to include more real-world data, both industry and academia will likely search for ways to balance model optimization with generalizability. This study provides a pragmatic approach to applying RCT-trained models to real-world data.

## Key Points

**Question:**

Are findings from prognostic models that are derived from randomized clinical trial (RCT) data generalizable to the real-world setting?

**Findings:**

In this cohort study of 2113 patients with metastatic castration-resistant prostate cancer, predictions from models trained on data from RCTs could not be generalized to the real-world setting with data from electronic health records (EHRs). Patient populations and feature availability differed substantially between the RCT–derived models and the EHR–derived models; the best-performing EHR–derived model included only 25 of the 101 variables included in the RCT–derived model and achieved adequate performance, as measured by integrated area under the curve.

**Meaning:**

This study’s findings indicate that prognostic models derived from RCT data provide insights into factors associated with mortality; however, the direct application of these models to real-world data requires local optimization, including feature reduction strategies and evaluation of algorithmic bias.

## Teaser

This cohort study assesses the performance of a prognostic survival model derived from randomized clinical trial data when the model is applied to real-world data from the electronic health records of patients with metastatic castration-resistant prostate cancer.

## Introduction

Randomized clinical trials (RCTs), although considered the criterion standard in evidence-based medicine, may be costly, and study populations may not be representative of patients in a real-world practice setting.^1^ Clinical trials are often conducted using protocols that differ substantially from processes used in routine care, and certain patient groups (eg, patients with multiple comorbidities) are often underrepresented in RCTs.^2,3,4,5,6^ It is estimated that only 5% of patients eligible for inclusion in an RCT are actually enrolled in one, calling into question the ability to generalize the results of RCTs to the population.^7^ Furthermore, clinical trials of patients with prostate cancer have been reported to underenroll individuals from racial and ethnic minority groups, particularly Black and African American individuals (in clinical trials of patients with metastatic disease) and Latino and Asian individuals (in all clinical trials).^8,9^ This issue highlights the increasing need to understand the applicability of interventions developed in RCTs to the real-world setting.

The availability of routinely collected clinical data through electronic health records (EHRs) offers opportunities to understand the generalizability of RCT findings to populations that better reflect real-world care.^10,11^ However, the information contained in EHRs is complex, contains diverse data types and formats, and is not organized for research purposes.^12^ The simple task of cohort identification (known as clinical phenotyping) can require advanced technologies and substantial resources to accurately classify populations into disease categories.^13^ This issue is particularly true for complex clinical subclassifications, such as the fatal form of prostate cancer known as metastatic castration-resistant prostate cancer (CRPC), for which specific diagnostic codes do not exist.^14,15,16^ A patient with CRPC is one who develops resistance to androgen ablation–depriving therapy, either chemical (hormone therapy) or surgical (orchiectomy).

Although a number of strategies exist for managing metastatic CRPC, the optimal selection and sequencing of these therapies is an active area of research.^17^ Better estimation of the individual risk profiles of patients with metastatic CRPC may facilitate more appropriate matching of patients to treatment pathways or clinical trials, enable personalization of second-line treatment regimens, and allow population-level evaluations. To address this need, the Dialogue for Reverse Engineering Assessments and Methods (DREAM) challenge for prostate cancer was held from March 16 to July 27, 2015. For this challenge, data from 4 phase 3 RCTs were made available in a crowdsourced contest to build the best survival model for patients with metastatic CRPC.^18^ The best-performing team used an ensemble of penalized Cox proportional hazards regression models to predict survival with a time-dependent integrated area under the curve (iAUC) of 0.768.

As regulatory changes require better use of real-world evidence for clinical assertions,^10^ there is an increasing need to understand the extent to which RCT evidence can be applied to routine clinical care. To this end, we developed a pragmatic approach to implement and evaluate a prognostic model for survival among patients with metastatic CRPC that was based on the DREAM challenge RCT-trained model and applied to real-world EHR data from patients who received treatment at a comprehensive cancer center. Our hypothesis was that the RCT model would have lower performance with real-world data and that local optimization of the RCT-trained model could improve performance.

## Methods

### Study Cohorts

The DREAM challenge RCT data were obtained through the Project Data Sphere online platform.^18^ This data set included the 3 RCTs of patients with metastatic CRPC that were used to train the models in the competition. Data from the fourth RCT, which was used to validate the models in the competition, were not available. Data included patient demographic characteristics, laboratory values, procedures, diagnoses, and drug prescriptions. This study followed the Strengthening the Reporting of Observational Studies in Epidemiology (STROBE) reporting guideline for cohort studies. The study was approved by the institutional review board of Stanford University with a waiver of informed consent because of its retrospective nature.

**Figure 1.**
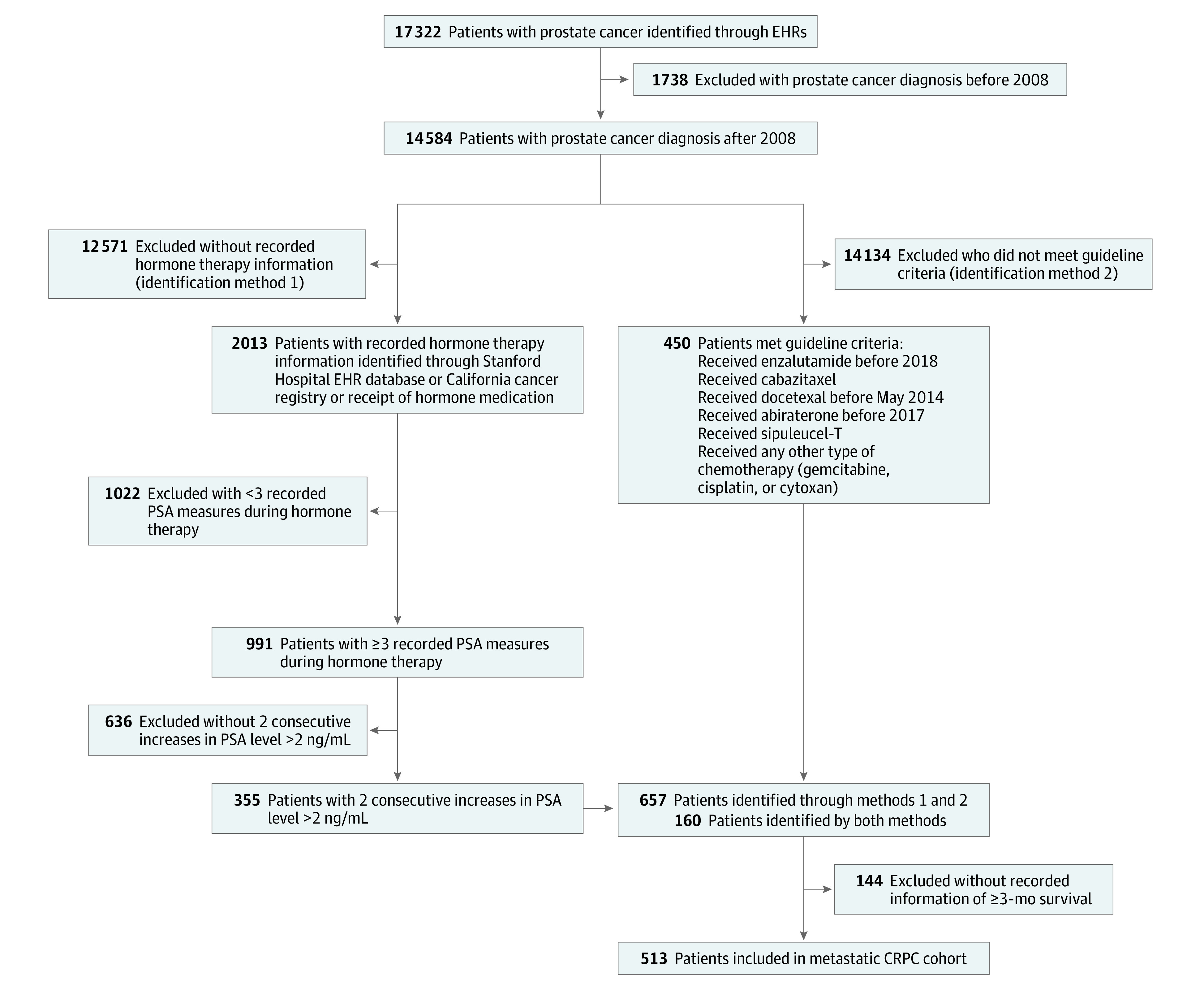
Study Flowchart CRPC indicates castration-resistant prostate cancer; EHR, electronic health record; and PSA, prostate-specific antigen.

Real-world data were obtained from the EHRs of a comprehensive cancer center at a tertiary care academic medical center; the process is described in detail elsewhere.^19^ Patients with prostate cancer between January 1, 2008, and December 31, 2019, were identified using diagnostic codes for prostate cancer from the *International Classification of Diseases, Ninth Revision, Clinical Modification* (codes 185 and 233.4) and the *International Classification of Diseases, Tenth Revision, Clinical Modification* (code C61). A total of 513 patients with metastatic CRPC were identified using 2 methods (Figure 1). For method 1, we identified patients receiving hormone therapy (eTable 1 in the Supplement). Patients were defined as having metastatic CRPC if, in addition to receiving first-line hormone therapy, they received cytotoxic chemotherapy, secondary hormone therapy, or immune modulatory therapy. For method 2, we identified patients with metastatic CRPC using patient-level prostate-specific antigen (PSA) trajectories to detect consecutive increases in PSA values during hormone therapy. An accepted definition of metastatic CRPC is the occurrence of 2 consecutive increases in serum PSA values while receiving hormone therapy.^20^ Patients were excluded if they did not have a recorded period during which they received hormone therapy (which is an indication of recurrent or metastatic disease) or if they did not have at least 3 recorded PSA values during receipt of hormone therapy. Hormone therapy was defined for every patient as the period of intake of specific drugs.

### Variables for Predictive Model

In the EHR data set, we identified 91 of the 101 variables included in the DREAM challenge RCT-derived model. Seven of the 91 variables were excluded because of missing data (eTable 2 and eTable 3 in the Supplement). Demographic variables were captured at the time of CRPC diagnosis.

For comorbidities, procedures, and drug prescriptions, data were captured 6 months before and 3 months after the date of CRPC diagnosis. Laboratory values were captured closest to the date of metastatic CRPC diagnosis within a 3-month period. Dates of death were collected from the California Cancer Registry, the Social Security Death Index, the internal Stanford University cancer registry, and the EHRs.

### Outcomes

The primary outcome of interest was the generalizability of the RCT-trained model when applied to real-world data, which was assessed by iAUC statistics. We also investigated racial disparities in the models’ training sets, which were conditional on the computed risk scores.^21^

A secondary outcome was survival from the time of development of metastatic CRPC, which was defined as the time from the date of CRPC diagnosis to the date of death. For patients who were alive at the end of the study or for whom no death information was available, we defined survival as the time from the date of CRPC diagnosis to the date of the last known follow-up (ie, right censoring).

### Predictive Model

To predict survival, we developed a penalized Cox proportional hazards regression model with elastic-net regularization based on the DREAM challenge RCT-derived model using 10-fold cross-validation. We first trained the model on the RCT data using the variables selected for the DREAM challenge model. Twelve skewed variables were log-transformed, and 0 values were replaced with the smallest non-0 value to ensure finite transformed values (eTable 4 in the Supplement). Next, we trained the model on the EHR data set. Missing values were imputed using a penalized Gaussian regression model based on nonmissing variables, as was performed in the DREAM challenge model.^18^ We then trained the model on 90% of the EHR data set.

To identify the variables most predictive of survival, we developed a correlation-based feature reduction strategy.^22^ We used a κ-means method to cluster variables into pairs with low, medium, and high correlation. Next, we used linear programming optimization techniques to remove sets of variables such that no highly correlated pairs remained, using the sum of the absolute correlation as the objective function. We then ranked the remaining variables according to their absolute correlation with the outcome (referred to as top variables). We explored the performance of models containing different numbers of top variables. In addition, we explored the accuracy of the model with the optimal features detected by the recursive feature elimination method. Based on results from the best-performing model, we divided the patients with the median model-predicted risk score into low-risk and high-risk mortality groups and assessed survival.

### Evaluation of Models and Disparities

To evaluate the models’ performance, we compared their mean 10-fold time-dependent iAUC between 6 and 30 months on 90% of the EHR data set. We also used the 10% of the data set (test set) that was not previously used for model development to assess the best-performing EHR model and compare it with the RCT-trained model.

For the 2 models trained on different data sets, we excluded patient race from the feature set to assess the impact of race disparities with regard to risk scores. For the DREAM challenge RCT model and the best-performing EHR model, we obtained risk scores generated for each patient-year. We plotted the function of mean comorbidity scores or PSA values against model risk score by different races.^21^

### Statistical Analysis

Patient demographic characteristics were compared using unpaired *t* tests for parametric data and analysis of variance for parametric data, and χ^2^ and Fisher exact tests were used for categorical variables. The Wilcoxon rank sum test for dependent samples was used to calculate the significance between the iAUCs. Kaplan-Meier estimators and log-rank statistics were used to assess survival. All statistical tests were 2-sided with a significance threshold of *P* ≤ .05. Analyses were performed using R software, version 4.0.3 (R Project for Statistical Computing). Data were analyzed from March 23, 2018, to October 22, 2020.

## Results

### Study Participants

Of the 17 322 patients with prostate cancer in the EHR, 513 patients (3.0%) with CRPC were identified (Figure 1), and no difference was observed in race distribution. A manual record review of 30 randomly selected patients found an accuracy of 83%. Table 1 shows patient demographic characteristics stratified by RCT cohort (1600 patients) and EHR cohort (513 patients).

**Table 1.** Characteristics of Participants With Metastatic Castration-Resistant Prostate Cancer^a^

Characteristic	No. (%)	
	EHR cohort	RCT cohort
Total participants, No.	513	1600
Age range at diagnosis, y		
18-54	32 (6.2)	69 (4.3)
55-64	95 (18.5)	432 (27.0)
65-74	195 (38.0)	711 (44.4)
≥75	191 (37.2)	388 (24.3)
BMI at diagnosis		
<18	15 (2.9)	2 (0.1)
18-24	174 (33.9)	373 (23.3)
25-35	293 (57.1)	1114 (69.6)
≥35	31 (6.0)	111 (6.9)
Insurance coverage at diagnosis		
Private	81 (15.8)	NA
Medicare	354 (69.0)	NA
Medicaid	26 (5.1)	NA
Unknown	52 (10.1)	NA
Race/ethnicity		
Non-Hispanic White	337 (65.7)	1390 (86.9)
Asian	88 (17.2)	41 (2.6)
Black/African American	24 (4.7)	74 (4.6)
Hispanic/Latino	42 (8.2)	14 (0.9)
Other^b^	22 (4.3)	81 (5.1)
Cancer stage at diagnosis		
I	12 (2.3)	NA
II	76 (14.8)	NA
III	14 (2.7)	NA
IV	117 (22.8)	NA
Unknown	294 (57.3)	1600 (100.0)
Tumor stage at diagnosis		
T1	103 (20.1)	40 (2.5)
T2	75 (14.6)	150 (9.4)
T3	33 (6.4)	157 (9.8)
T4	10 (2.0)	60 (3.7)
TX	33 (6.4)	36 (2.3)
Unknown	259 (50.5)	1157 (72.3)
Charlson Comorbidity Index score within 2 y before diagnosis, mean (SD)	2.5. (2.6)	1.6 (1.8)
Receipt of docetaxel therapy at diagnosis		
Yes	53 (10.3)	1586 (99.1)
No	460 (89.7)	14 (0.9)
Log-transformed PSA value at diagnosis, mean (SD)	2.7. (1.6)	4.4. (1.6)

Abbreviations: BMI, body mass index (calculated as weight in kilograms divided by height in meters squared); EHR, electronic health record; NA, not available; PSA, prostate-specific antigen; RCT, randomized clinical trial.

^a^ All *P* values were <.001.

^b^ Other race category includes Pacific Islander and American Indian.

The RCT cohort included a smaller proportion of participants who were Hispanic (14 patients [0.9%] vs 42 patients [8.2%]), Asian (41 patients [2.6%] vs 88 patients [17.2%]), and older than 75 years (388 patients [24.3%] vs 191 patients [37.2%]) compared with the EHR cohort. Participants in the RCT cohort also had fewer comorbidities (mean [SD], 1.6 [1.8] comorbidities vs 2.5 [2.6] comorbidities) compared with those in the EHR cohort. The number of encounters did not differ by race for all face-to-face health care encounters (eg, mean [SD], 67.97 [52.27] encounters among non-Hispanic White patients vs 77.07 [57.27] encounters among Hispanic or Latino patients; *P* = .46), primary care encounters (eg, mean [SD], 3.56 [7.78] encounters among non-Hispanic White patients vs 4.93 [10.14] encounters among Hispanic or Latino patients; *P* = .56), or oncological encounters (eg, mean [SD], 23.73 [23.88] encounters among non-Hispanic White patients vs 25.40 [21.05] encounters among Hispanic or Latino patients; *P* = .46) (eTable 6 in the Supplement).

### DREAM Challenge RCT-Derived Model

Of the 101 variables used in the RCT-derived model, 10 were not available in the EHR data set, 3 of which were among the top 10 features in the DREAM challenge RCT model. These variables included the Eastern Cooperative Oncology Group score, which measures functional status, and 2 variables that specify metastases in different sites (liver and lymphatic system). Among the remaining 91 variables, 7 were not consistently captured in the EHRs and were removed because of missingness (eTable 3 in the Supplement). Using the remaining 84 variables and imputing the missing variables, the original DREAM challenge RCT-trained model achieved an average 10-fold mean (SD) iAUC of 0.722 (0.118) in the EHR data set compared with the iAUC of 0.768 achieved in the original DREAM challenge test data set.

For local optimization, the model was retrained using the 84 available variables in the EHR data set, achieving a mean (SD) iAUC of 0.762 (0.106). Using our feature reduction strategy, we removed 24 correlated variables to develop a model with 60 variables. Additional feature optimization using filtering feature selection produced multiple models containing between 10 and 60 features, with mean (SD) iAUC values between 0.775 (0.098) and 0.792 (0.097). Using recursive feature elimination, our best-performing EHR model included 25 variables and achieved a mean (SD) iAUC of 0.792 (0.097) (Table 2). To validate the best-performing model trained on EHR data, we applied the model to the validation data set that was not previously used for model development, achieving an iAUC of 0.596 compared with an iAUC of 0.398 for the RCT-trained model (*P* < .001).

**Table 2.** Model Performance for Predicting Survival in the Electronic Health Record Cohort

Training cohort	Variables, No.	iAUC, mean (SD)^a^
RCT data^b^	101	0.722 (0.118)
EHR data with all variables available	84	0.762 (0.106)
EHR data with all independent variables (no covariates)	60	0.775 (0.098)
EHR data with top 25 variables selected by RFE^c^	25	0.792 (0.097)
EHR data with top 15 variables^c^	15	0.785 (0.098)
EHR data with top 10 variables^c^	10	0.779 (0.099)

Abbreviations: EHR, electronic health record; iAUC, integrated area under the curve; RCT, randomized clinical trial; RFE, recursive feature elimination.

^a^ Mean (SD) iAUC from the 10-fold validation on 90% of the EHR data set.

^b^ Among the 101 RCT variables, 17 were not available in the EHR data set because they are not routinely collected. Their missing values were imputed using a penalized Gaussian regression model based on nonmissing variables. Other partially missing variables were also imputed.

^c^ Top variables are those with the highest absolute correlation with the outcome.

Among the top 10 predictive features (eTable 5 in the Supplement) in the EHR-trained models, 5 were not among the top 10 features in the DREAM challenge RCT-derived model. These features were lymphocytes, the ratio of neutrophil level to blood cells, spinal cord compression, body mass index (calculated as weight in kilograms divided by height in meters squared), and pulse.

**Figure 2.**
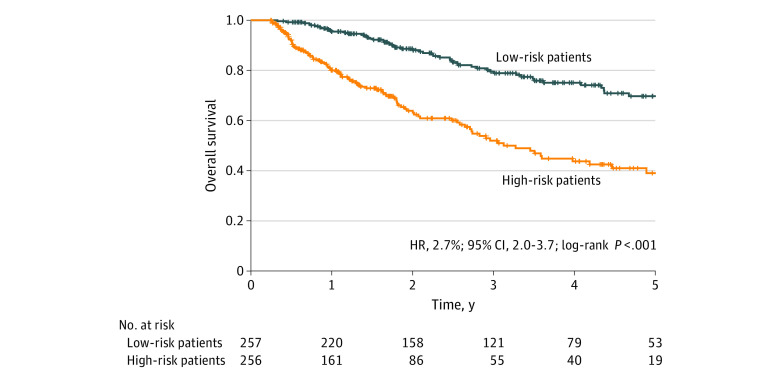
Five-Year Overall Survival Among Patients in EHR Cohort, Stratified by Risk Group Kaplan-Meier plot of 5-year survival probability for low-risk patients vs high-risk patients with metastatic CRPC in the EHR cohort. The stratification was within the median of model-predicted risk scores based on results from the best-performing predictive model trained with data from the EHR cohort. CRPC indicates castration-resistant prostate cancer; EHR, electronic health record; HR, hazard ratio.

Using the best-performing EHR-trained model, we classified patients into high-risk and low-risk mortality groups using a median-split method (Figure 2). The difference in 5-year overall survival between the high-risk group (256 patients) and the low-risk group (257 patients) was significant (hazard ratio, 2.7; 95% CI, 2.0-3.7; *P* < .001).

**Figure 3.**
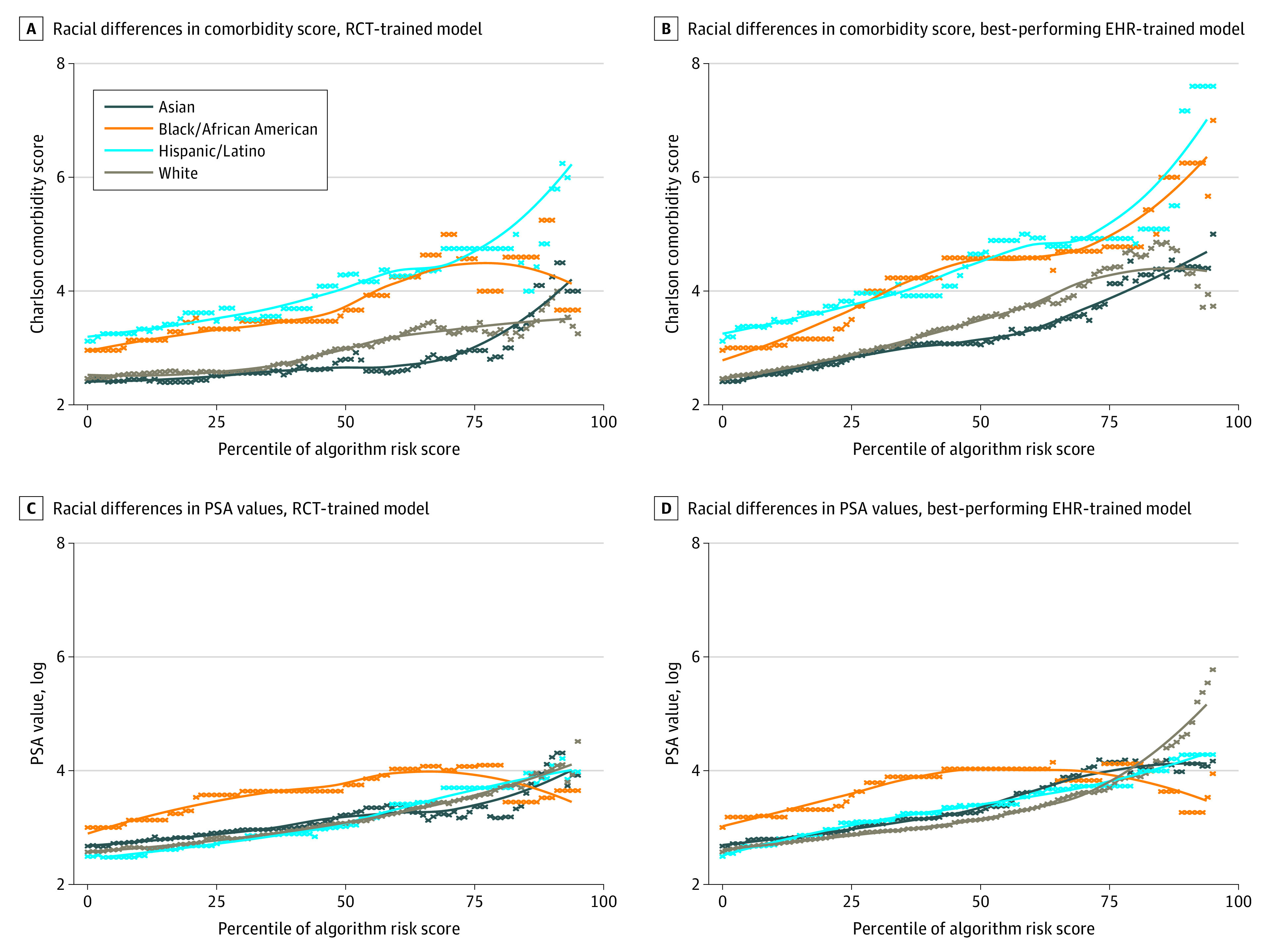
Impact of Predicted Survival Risk Scores for Charlson Comorbidity Scores and PSA Values, Stratified by Model Training Data Set and Patient Race The Dialogue for Reverse Engineering Assessments and Methods (DREAM) model was used to train data from randomized clinical trials. EHR indicates electronic health record; PSA, prostate-specific antigen; and RCT, randomized clinical trial. A, Racial differences in comorbidity score, RCT-trained model. B, Racial differences in comorbidity score, best-performing EHR-trained model. C, Racial differences in PSA values, RCT-trained model. D, Racial differences in PSA values, best-performing EHR-trained model.

Patient race was not significant as a predictor of survival in either the RCT or EHR cohort (eFigure in the Supplement). To quantify the impact of race disparities with regard to risk scores, we calculated algorithmic risk scores by comorbidities and PSA values, stratified by race (Figure 3). These calculations indicated that Black and Hispanic participants required a larger number of chronic conditions and higher PSA values than White and Asian patients to receive the same algorithmic-derived risk score.

## Discussion

Translating RCT-derived clinical data to real-world settings is a priority for advancing clinical practice, as exemplified by the 21st Century Cures Act.^10^ In this study, we applied an RCT-trained prognostic model for metastatic CRPC to real-world data and provided a framework for evidence translation. The direct application of the RCT-trained model to real-world data did not perform well; substantial differences in patient demographic characteristics and disease severity are likely factors underlying the model’s low performance. However, through local model optimization, including feature reduction strategies, we highlighted the ways in which the RCT findings can produce a model using real-world data that has good discrimination abilities. These successful results have the potential to change the outlook on the secondary use of both RCT and EHR data, increasing the ability to understand the impact of clinical therapies for a broader and more generalizable population seeking routine clinical care for the disease being studied.

We developed a prognostic model based on the DREAM challenge RCT-trained model, which accurately discriminated between low-risk and high-risk patients. The top predictive features from our model differed slightly from the RCT model based on the availability of specific variables in the EHRs. In our model, the presence of high alkaline phosphatase levels, high aspartate aminotransferase levels, and high PSA values at the time of CRPC diagnosis were predictive of a worse prognosis. These variables were also identified by the DREAM challenge’s best-performing team.^18^ While many of these variables have been previously reported to have an association with survival in laboratory and clinical trials, our work highlights the complexity of translating RCT-derived models to the real-world setting, as data on many of the prognostic factors used in the RCT-derived model are not routinely collected in real-world settings.

The EHR-trained models that were developed using all available features had good discrimination in identifying patients with metastatic CRPC with a high risk of mortality within 5 years. Data collected in RCTs are likely superior to data collected in real-world settings with regard to statistical noise, accuracy, and missingness, as trained administrators collected these data using protocol guidelines. Furthermore, RCT data may better capture patient-reported outcomes and survival, whereas advanced technologies are needed to identify and extract this information in EHRs.^23,24,25^ Hence, the accuracy of the EHR model improved as feature reduction strategies were applied; the optimal model included only 25 of the 101 features included in the RCT-derived model. These feature reduction strategies are important when using real-world data because data collection is expensive for the health care system, requiring time as well as computational and financial resources, particularly if many of the identified features do not have substantial implications for model performance. Therefore, developing a model with a minimal number of features allows us to be more realistic about its implementation in a hospital setting.^26^ The extent of EHR data missingness highlights the importance of feature reduction strategies, and this study provides a framework to account for the missingness and inaccuracy of real-world data to produce relevant prognostic models at the point of care.

Consistent with existing literature,^2,3,4,8^ we found substantial differences in racial characteristics between the RCT and EHR cohorts. Most patients in the RCT cohort were White, and few patients from racial or ethnic minority groups were included. This composition differed from the population seeking care in the routine setting, which had substantially higher proportions of Black, Hispanic, and Asian patients. Across the 4 RCTs, fewer than 1% of study participants were Hispanic or Latino. This finding suggests that the RCT population is not representative of the average patient seeking care at a comprehensive cancer center, which has been noted in other RCTs of patients with prostate cancer.^8^ However, because potential biases in the collection of race information could exist, both in the original RCT data and in our EHR data, robust examination of bias and algorithmic fairness is warranted when either type of data is used for predictive models.^27^ By extending the results of RCT models to EHRs, one can optimize the inclusion of diverse populations who are eligible for an intervention but may not meet the strict inclusion criteria of an RCT. This expansion to diverse populations may help mitigate potential bias.^28^

The RCT data also differed from real-world data with regard to other patient demographic and clinical characteristics. The RCT populations in the DREAM challenge included men who were predominantly aged 65 to 75 years, with substantially fewer patients older than 75 years compared with the real-world setting, which is similar to other findings.^4^ Patients included in the RCT cohort also had a lower number of comorbidities compared with patients in the EHR cohort. Because increasing age and the complexity of a patient’s condition are associated with survival,^29^ our study highlights the importance of optimizing RCT-derived models for real-world settings, which include patients with more complex conditions.

The goal of all clinical predictive models is to help clinical decision-making; hence, it is important to investigate potential bias before deploying models at the point of care. Racial bias in predictive models is an emerging issue, and research suggests that some models may be associated with perpetuating racial disparities in health care.^8,21^ This finding is particularly true when training data are not representative of the population to which the models will be applied. To investigate bias in the metastatic CRPC prognostic models, we evaluated the association between the model-derived risk scores, Charlson Comorbidity Index scores, and PSA values. In both data sets, our results indicated that Black patients needed higher Charlson Comorbidity Index scores and PSA values to receive a risk score that was equivalent to that of non-Hispanic White patients. In the EHR cohort, race and ethnicity were not associated with a higher number of health care encounters, so other factors are likely associated with these biases. When models are deployed at the point of care, such biases can inform resource allocation. This study’s findings highlight the necessity of evaluating population representativeness and potential biases.^30^ The transparency in such information provides the end user with an opportunity to mitigate such biases and appropriately deploy models across populations.

In the shifting landscape of clinical criterion standards, the increasing use of real-world data is likely.^10^ Emerging findings highlight the need to understand the challenges and opportunities of using these data for evidence generation,^31^ which is important for future pragmatic approaches toward clinical trials. We present a timely and innovative approach to validate criterion-standard evidence from RCTs using routinely collected clinical data that may augment the implementation of data-driven decisions at the point of care. Identifying the appropriate treatment for each patient is important, particularly as patients’ values and shared decision-making become a greater focus in clinical care; providing tools to better counsel patients on their potential individual risk is needed. While RCTs are critiqued for being nongeneralizable and expensive, they are still and will likely remain the criterion standard for clinical assertions. Real-world data can be used to augment and validate RCT findings, as we have done in this study, and can expand the application of RCT-based models to broader patient populations.

### Limitations

This study has several limitations. First, in real-world settings, patients seek care at their convenience; this process may differ substantially from those used in RCTs, which adhere to strict protocols, and may impact patient outcomes. However, real-world data better reflect practice standards and the ways in which the general patient population seeks care. Second, there are gaps of information regarding the receipt of intermittent hormone therapy, and the exact dates of a given prescription may not always be complete. However, linking EHRs to registry data can fill these gaps. Despite these shortcomings, this study provides guidance for the use of RCT-derived data in a real-world setting.

## Conclusions

In this study, we present a framework to apply clinical trial findings more broadly to patients with the disease being studied; this framework focuses on local optimization of an RCT-based predictive model for real-world data. We found that a machine learning model trained on an RCT data set did not perform well when applied directly to real-world data. However, through feature reduction strategies, we developed a model that was more attuned to our health care setting and that had better diversity and a feasible selection of top features. The findings of any prognostic model are only useful to the patient and clinician if they can be implemented at the bedside. While RCTs may be superior for model building with regard to data quality and integrity, it is feasible to streamline the translational research pipeline using real-world data to better inform decision-making. More research is warranted to understand the ways in which our findings can be translated into a clinical decision aid that is personalized to an individual patient’s health record.

## Notes

Supplement. **eTable 1.** Medications Used to Identify Receipt of Hormone Therapy **eTable 2.** Variables From the DREAM Challenge RCT-Trained Model Also Identified in EHR Data **eTable 3.** Variables From the DREAM Challenge RCT-Trained Model Included in EHR-Trained Models **eTable 4.** Log-Transformed Variables **eTable 5.** Coefficients of the Top 10 Variables in the Best-Performing EHR-Trained Models **eTable 6.** Number of Encounters in the Year After Metastatic CRPC Diagnosis, Stratified by Patient Race **eFigure.** 5-Year Overall Survival Among Patients with Metastatic CRPC in the EHR Cohort, Stratified by Patient Race
