# Supplementary material for: Assessment of a Clinical Trial–Derived Survival Model in Patients With Metastatic Castration-Resistant Prostate Cancer
Source: JAMA Netw Open. 2021 Jan 22;4(1):e2031730. doi: 10.1001/jamanetworkopen.2020.31730 (PMC7823224; doi:10.1001/jamanetworkopen.2020.31730)
Supplement: Supplement. — eTable 1. Medications Used to Identify Receipt of Hormone Therapy eTable 2. Variables From the DREAM Challenge RCT-Trained Model Also Identified in EHR Data eTable 3. Variables From the DREAM Challenge RCT-Trained Model Included in EHR-Trained Models eTable 4. Log-Transformed Variables eTable 5. Coefficients of the Top 10 Variables in the Best-Performing EHR-Trained Models eTable 6. Number of Encounters in the Year After Metastatic CRPC Diagnosis, Stratified by Patient Race eFigure. 5-Year Overall Survival Among Patients with Metastatic CRPC in the EHR Cohort, Stratified by Patient Race [file jamanetwopen-e2031730-s001.pdf]

Supplementary Online Content

Coquet J, Bievre N, Billaut V, et al. Assessment of a clinical trial–derived survival model in patients with metastatic castration-resistant prostate cancer. *JAMA Netw Open*. 2021;4(1):e2031730. doi:10.1001/jamanetworkopen.2020.31730

eTable 1. Medications Used to Identify Receipt of Hormone Therapy

eTable 2. Variables From the DREAM Challenge RCT-Trained Model Also Identified in EHR Data

eTable 3. Variables From the DREAM Challenge RCT-Trained Model Included in EHR-Trained Models

eTable 4. Log-Transformed Variables

eTable 5. Coefficients of the Top 10 Variables in the Best-Performing EHR-Trained Models

eTable 6. Number of Encounters in the Year After Metastatic CRPC Diagnosis, Stratified by Patient Race

eFigure. 5-Year Overall Survival Among Patients with Metastatic CRPC in the EHR Cohort, Stratified by Patient Race

This supplementary material has been provided by the authors to give readers additional information about their work.

© 2021 Coquet J et al. *JAMA Network Open*.

eTable 1. Medications Used to Identify Receipt of Hormone Therapy

**Generic Name**	**RxNorm**
Flutamide	4508
Ketoconazole	6135
Cyproterone Acetate	22054
Nilutamide	31805
Triptorelin	38782
Leuprolide	42375
Goserelin	50610
Histrelin	50975
Zoladex	58328
Bicalutamide	83008
Casodex	151495
Nizoral	202692
Goserelin Acetate	203146
Leuprolide Acetate	203217
Leuprorelin Acetate	203217
Lupron	203852
Nilandron	218741
Triptorelin Pamoate	338529
Eligard	352619
Degarelix	475230
Vantas	606382
Firmagon	858122
Trelstar	905054
Histrelin Acetate	1294622

© 2021 Coquet J et al. *JAMA Network Open*.

eTable 2. Variables From the DREAM Challenge RCT-Trained Model Also Identified in EHR Data

Rank in the RCT-trained Model	Variable	Type	Missingness in EHRs data
1	Liver Lesion(s)	Binary	100.0%
2	Patient Performance Status	Categorical	100.0%
3	Target Lesion(s)	Binary	100.0%
4	Alkaline Phosphatase	Numerical	18.5%
5	Prostate Specific Antigen	Numerical	41.3%
6	Hemoglobin	Numerical	18.1%
7	Hematocrit	Numerical	18.1%
8	Ratio Lymphocyte / Leucocyte	Numerical	20.1%
9	Aspartate Aminotransferase	Numerical	18.3%
10	Albumin	Numerical	18.3%
11	Red Blood Cells	Numerical	18.1%
12	Systolic Blood Pressure	Numerical	7.8%
13	Lactate Dehydrogenase	Numerical	88.1%
14	Body Mass Index	Numerical	12.1%
15	Diastolic Blood Pressure	Numerical	7.8%
16	Blood Urea Nitrogen	Numerical	20.3%
17	Alanine Transaminase	Numerical	19.3%
18	Calculated Creatinine Clearance	Numerical	100.0%
19	Sodium	Numerical	20.3%
20	Creatinine Clearance	Numerical	100.0%
21	Glucose	Numerical	20.3%
22	Weight	Numerical	12.3%
23	Specific Gravity	Numerical	100.0%
24	Total Protein	Numerical	18.5%
25	Calcium	Numerical	20.7%
26	Total Bilirubin	Numerical	29.8%
27	Creatinine	Numerical	100.0%
28	Ratio Neutrophil / Leucocyte	Numerical	20.1%
29	White Blood Cells	Numerical	18.1%
30	Neutrophils	Numerical	20.1%
31	Ratio Monocyte / Leucocyte	Numerical	19.5%
32	Pulse	Numerical	7.2%
33	Potatium	Numerical	20.3%
34	Height	Numerical	12.5%

© 2021 Coquet J et al. *JAMA Network Open*.

35	Monocyte	Numerical	19.5%
36	Race Category: White	Binary	0.0%
37	Patient received analgesics	Binary	2.9%
38	Ratio Basophils / Leucocyte	Numerical	19.5%
39	Patient received anti androgens	Binary	2.9%
40	Platelet Count	Numerical	17.9%
41	Eosinophil	Numerical	19.5%
42	Ratio Eosinophil / Leucocyte	Numerical	19.5%
43	Age group	Categorical	0.0%
44	If the patient is from East Europe	Binary	0.0%
45	Lymphocytes	Numerical	20.1%
46	Lymph node Lesion(s)	Binary	100.0%
47	Testosterone	Numerical	100.0%
48	Patient received gomadotropin	Binary	2.9%
49	If the patient has a medical history of Cardiac disorders	Binary	0.2%
50	If the patient has a medical history of Vascular disorders	Binary	0.2%
51	If the patient has a medical history of Myocardial Infarction	Binary	0.2%
52	If the patient received Prostatectomy	Binary	3.9%
53	Phosphorus	Numerical	84.4%
54	Prostate Lesion(s)	Binary	100.0%
55	If the patient received Radiotherapy	Binary	0.2%
56	If the patient received Bisphosponate	Binary	2.9%
57	If the patient received a Bilateral Orchidectomy	Binary	3.9%
58	If the patient has a medical history of Psychiatric disorders	Binary	0.2%
59	If the patient is from West Europe	Binary	0.0%
60	If the patient has a Medical History of Congestive Heart Failure	Binary	0.2%
61	Magnesium	Numerical	76.6%
62	If the patient received Bisphosponate Ace Inhibitors	Binary	2.9%
63	If the patient received Orchidectomy (including bilateral)	Binary	3.9%
64	If the patient has a Medical History of Pulmonary Embolism	Binary	0.2%
65	Lung Lesion(s)	Binary	100.0%
66	If the patient has a Medical History of Spinal Cord Compression	Binary	0.2%
67	If the patient received Bilateral Lymphadenectomy	Binary	3.9%

© 2021 Coquet J et al. *JAMA Network Open*.

68	Pleura Lesion(s)	Binary	100.0%
69	If the patient has a Medical History of Metabolism & Nutrition disorders	Binary	0.2%
70	If the patient has a Medical History of Diabetes	Binary	0.2%
71	If the patient has a Medical History of Resperatory, Thoracic & Mediastinal disorders	Binary	0.2%
72	If the patient received Glucocorticoids	Binary	2.9%
73	If the patient received Imidazole	Binary	2.9%
74	If the patient has a Medical History of Hepatobiliary disorders	Binary	0.2%
75	If the patient has a Medical History of Chronic Obstructive Pulmonary disease	Binary	0.2%
76	If the patient received Estrogens	Binary	2.9%
77	If the patient has a Medical History of Renal & Urinary disorders	Binary	0.2%
78	If the patient has a Medical History of Gastrointestinal disorders	Binary	0.2%
79	If the patient has a Medical History of Infections & Infestations	Binary	0.2%
80	If the patient has a Medical History of Injury, Poison & Procedural	Binary	0.2%
81	If the patient received HMG-CoA Reductase Inhibitors	Binary	2.9%
82	If the patient received Corticosteroid	Binary	2.9%
83	If the patient has a Medical History of Ear & Labyrinth disorders	Binary	0.2%
84	If the patient is from North America	Binary	0.0%
85	Race Category: Other	Binary	0.0%
86	If the patient is from South America	Binary	0.0%
87	If the patient has a Medical History of Immune System disorders	Binary	0.2%
88	If the patient has a Medical History of Cerebrovascular Accident (Hemorrhagic and/or Ischemic)	Binary	0.2%
89	If the patient has a Medical History of Endocrine disorders	Binary	0.2%
90	If the patient has a Medical History of Skin & Subcutaneous tissue disorders	Binary	0.2%
91	If the patient has a Medical History of Blood & Lymphatic system disorders	Binary	0.2%
92	Kidney Lesion(s)	Numerical	100.0%
93	If the patient has a Medical History of Congenital, Familial & Genetic disorders	Binary	0.2%
94	Race Category: Asian	Binary	0.0%
95	If the patient has a Medical History of	Binary	0.2%

© 2021 Coquet J et al. *JAMA Network Open*.

	investigations		
96	If the patient received Anti-Estrogens	Binary	2.9%
97	If the patient has a Medical History of Deep Venous Thrombosis	Binary	0.2%
98	If the patient received Beta Blocking Agents	Binary	2.9%
99	Race Category: Black	Binary	0.0%
100	Other Lesion(s)	Binary	100.0%
101	If the patient is from Asia	Binary	0.0%

Abbreviations: EHRs, Electronic Health Records; RCT, Randomized Clinical Trial.

© 2021 Coquet J et al. *JAMA Network Open*.

eTable 3. Variables From the DREAM Challenge RCT-Trained Model Included in EHR-Trained Models

Variable	All independent variables (no covariates)	Top 25 variables selected by RFE	Top 15 variables	Top 10 variables
Albumin	Y	Y	Y	Y
Alkaline Phosphatase	Y	Y	Y	Y
Aspartate Aminotransferase	Y	Y	Y	Y
Body Mass Index	Y	Y	Y	Y
Lymphocytes	Y	Y	Y	Y
If the patient has a medical history of Cardiac disorders	Y	Y	Y	Y
If the patient has a Medical History of Resperatory, Thoracic & Mediastinal disorders	Y	Y	Y	Y
If the patient received Radiotherapy	Y	Y	Y	Y
Prostate Specific Antigen	Y	Y	Y	Y
If the patient has a Medical History of Spinal Cord Compression	Y	Y	Y	Y
Patient received analgesics	Y	Y	Y	N
If the patient has a Medical History of Congestive Heart Failure	Y	Y	Y	N
Hemoglobin	Y	Y	Y	N
Pulse	Y	Y	Y	N
Systolic Blood Pressure	Y	Y	Y	N
Diastolic Blood Pressure	Y	Y	N	N
Glucose	Y	Y	N	N
If the patient received Glucocorticoids	Y	Y	N	N
If the patient has a Medical History of Ear & Labyrinth disorders	Y	Y	N	N
If the patient has a Medical History of Immune System disorders	Y	Y	N	N
If the patient has a Medical History of Infections & Infestations	Y	Y	N	N
If the patient has a Medical History of Renal & Urinary disorders	Y	Y	N	N
If the patient has a Medical History of Skin & Subcutaneous tissue disorders	Y	Y	N	N
If the patient has a medical history of Myocardial Infarction	Y	Y	N	N
Ratio Neutrophil / Leucocyte	Y	Y	N	N

© 2021 Coquet J et al. *JAMA Network Open*.

If the patient received Bisphosponate Ace Inhibitors	Y	N	N	N
Age group	Y	N	N	N
Alanine Transaminase	Y	N	N	N
Patient received anti androgens	Y	N	N	N
If the patient received Anti-Estrogens	Y	N	N	N
If the patient received Beta Blocking Agents	Y	N	N	N
If the patient received Bisphosponate	Y	N	N	N
Blood Urea Nitrogen	Y	N	N	N
Calcium	Y	N	N	N
If the patient has a Medical History of Cerebrovascular Accident (Hemorrhagic and/or Ischemic)	Y	N	N	N
If the patient has a Medical History of Chronic Obstructive Pulmonary disease	Y	N	N	N
If the patient has a Medical History of Deep Venous Thrombosis	Y	N	N	N
Ratio Eosinophil / Leucocyte	Y	N	N	N
If the patient received Estrogens	Y	N	N	N
Patient received gomadotropin	Y	N	N	N
Height	Y	N	N	N
If the patient received HMG-CoA Reductase Inhibitors	Y	N	N	N
If the patient received Imidazole	Y	N	N	N
If the patient has a Medical History of Blood & Lymphatic system disorders	Y	N	N	N
If the patient has a Medical History of Congenital	Y	N	N	N
If the patient has a Medical History of Endocrine disorders	Y	N	N	N
If the patient has a Medical History of Injury, Poison & Procedural	Y	N	N	N
If the patient has a Medical History of investigations	Y	N	N	N
If the patient has a medical history of Psychiatric disorders	Y	N	N	N
Monocyte	Y	N	N	N
Sodium	Y	N	N	N
Neutrophils	Y	N	N	N
If the patient received Orchidectomy (including bilateral)	Y	N	N	N

© 2021 Coquet J et al. *JAMA Network Open*.

Platelet Count	Y	N	N	N
Potatium	Y	N	N	N
If the patient received Prostatectomy	Y	N	N	N
If the patient has a Medical History of Pulmonary Embolism	Y	N	N	N
Total Bilirubin	Y	N	N	N
Total Protein	Y	N	N	N
White Blood Cells	Y	N	N	N

Abbreviations: EHRs, Electronic Health Records; RCT, Randomized Clinical Trial; RFE, Recursive Feature Elimination.

© 2021 Coquet J et al. *JAMA Network Open*.

eTable 4. Log-Transformed Variables

**Variables**
Alanine transaminase
Alkaline phosphatase
Aspartate aminotransferase
Blood urea nitrogen
Glucose
Lymphocytes
Magnesium
Neutrophils
Phosphorus
Prostate specific antigen
Total bilirubin
White blood cells

© 2021 Coquet J et al. *JAMA Network Open*.

eTable 5. Coefficients of the Top 10 Variables in the Best-Performing EHR-Trained Models

Rank	Variable	Weight	HR	Top 10 DREAM RCT model
1	Lymphocytes 10^9/L	-0.163	0.850	N
2	Alkaline Phosphatase U/L	0.133	1.142	Y
3	Aspartate Aminotransferase U/L	0.127	1.135	Y
4	Prostate Specific Antigen Ng/Ml (PSA)	0.100	1.105	Y
5	Ratio of Neutrophil Level Per Blood Cell	0.084	1.087	N
6	Spinal Cord Compression	0.083	1.087	N
7	Baseline Body Mass Index Kg/M2 (BMI)	-0.082	0.922	N
8	Hemoglobin G/Dl	-0.078	0.925	Y
9	Pulse	0.074	1.077	N
10	Albumin G/L	-0.074	0.929	Y

Abbreviations: HR, Hazard Ratio.

© 2021 Coquet J et al. *JAMA Network Open*.

eTable 6. Number of Encounters in the Year After Metastatic CRPC Diagnosis, Stratified by Patient Race

	**Non-Hispanic White**	**Asian**	**Black**	**Hispanic/ Latino**	**Other**	**p**
**All types**	67.97 ± 52.27	79.41 ± 70.67	74.92 ± 50.27	77.07 ± 57.27	67.95 ± 55.50	0.46
**Oncology/ Urology**	23.73 ± 23.88	29.41 ± 35.03	27.88 ± 36.50	25.40 ± 21.05	27.27 ± 24.61	0.46
**Primary Care**	3.56 ± 7.78	5.06 ± 11.37	2.88 ± 8.15	4.93 ± 10.14	3.73 ± 8.57	0.56

© 2021 Coquet J et al. *JAMA Network Open*.

eFigure. 5-Year Overall Survival Among Patients with Metastatic CRPC in the EHR Cohort, Stratified by Patient Race

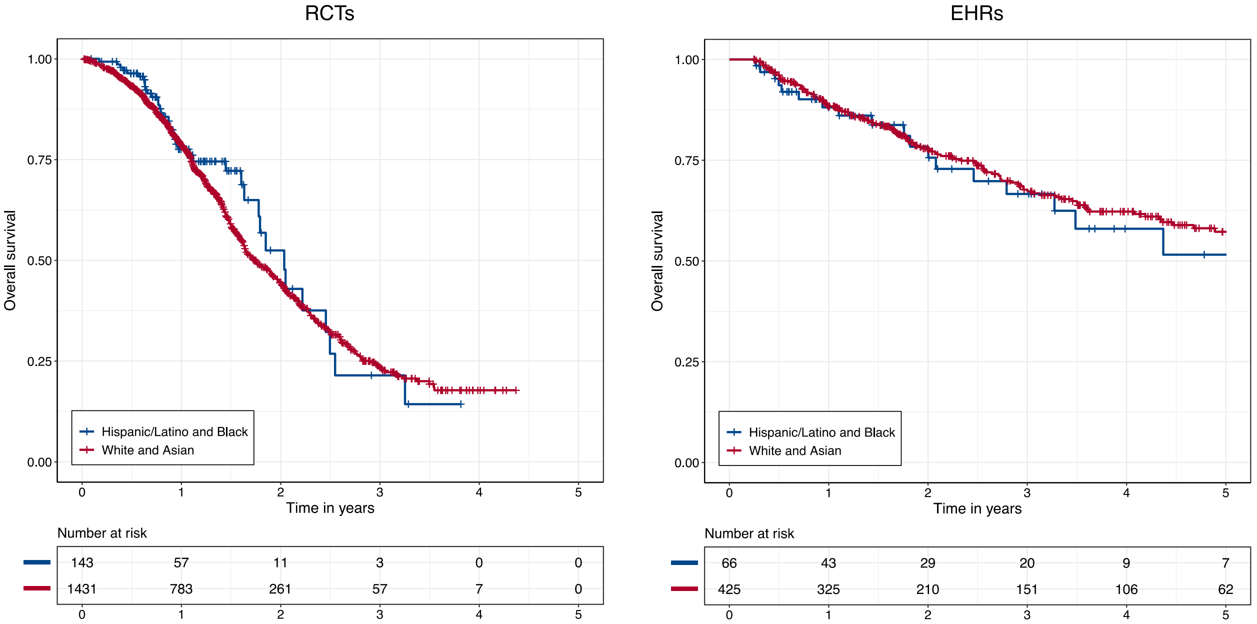

Kaplan-Meier plot of survival probability for White-Asian (red) and Hispanic/Latino-Black (blue) of the RCTs and the EHRs cohorts (P=.3 and P=.8, respectively).

© 2021 Coquet J et al. *JAMA Network Open*.
